# Proteomes of Animal Oocytes: What Can We Learn for Human Oocytes in the *In Vitro* Fertilization Programme?

**DOI:** 10.1155/2014/856907

**Published:** 2014-04-03

**Authors:** Irma Virant-Klun, Jeroen Krijgsveld

**Affiliations:** ^1^Reproductive Unit, Department of Obstetrics and Gynaecology, University Medical Centre Ljubljana, Slajmerjeva 3, 1000 Ljubljana, Slovenia; ^2^European Molecular Biology Laboratory, Meyerhofstraße 1, 69117 Heidelberg, Germany

## Abstract

Oocytes are crucial cells for mammalian reproduction, yet the molecular principles underlying oocyte development are only partially understood. Therefore, contemporary proteomic approaches have been used increasingly to provide new insights into oocyte quality and maturation in various species such as mouse, pig, and cow. Especially, animal studies have helped in elucidating the molecular status of oocytes during *in vitro* maturation and other procedures of assisted reproduction. The aim of this review is to summarize the literature on mammalian oocyte proteome and secretome research in the light of natural and assisted reproduction and on lessons to be learned for human oocytes, which have so far remained inaccessible for proteome analysis.

## 1. Introduction

The oocyte is one of the crucial cells in the live organisms, which enables the reproduction and continuation of the species. Because the main role of an oocyte is to be fertilized and to grow further into a fully functional organism, it needs to regulate many different cellular and developmental processes, such as cellular metabolism, cell cycle progression, fertilization, activation of zygotic transcription, embryo development, activation of the embryonic genome, and formation of embryonic axes [1–6]. During oocyte growth and maturation a variety of maternally transcribed mRNAs accumulate, representing the maternal contribution to the oocyte and, consequently, the newly fertilized oocyte, zygote, and early embryo [7–9]. The majority of these mRNAs are stored in message ribonucleoprotein (mRNP) complexes and are only translated when needed at specific stages of maturation [10]. In addition, they can be localized within a specific region of the cytoplasm or as being dispersed within the cytoplasm of the entire oocyte. Early embryonic development before the activation of embryonal genome is directed by maternal mRNAs expressed in oocytes and stored in mRNPs and occurs in the mid two-cell stage in the mouse, the four-cell stage in the pig, the eight-cell stage in the sheep, and between the four- and eight-cell stages in humans [11]. The real proportion of genes expressed in oocytes is still poorly understood but it is clear that translational activity and its regulation are crucial for oocyte development and maturation [12].

**Figure 1:**
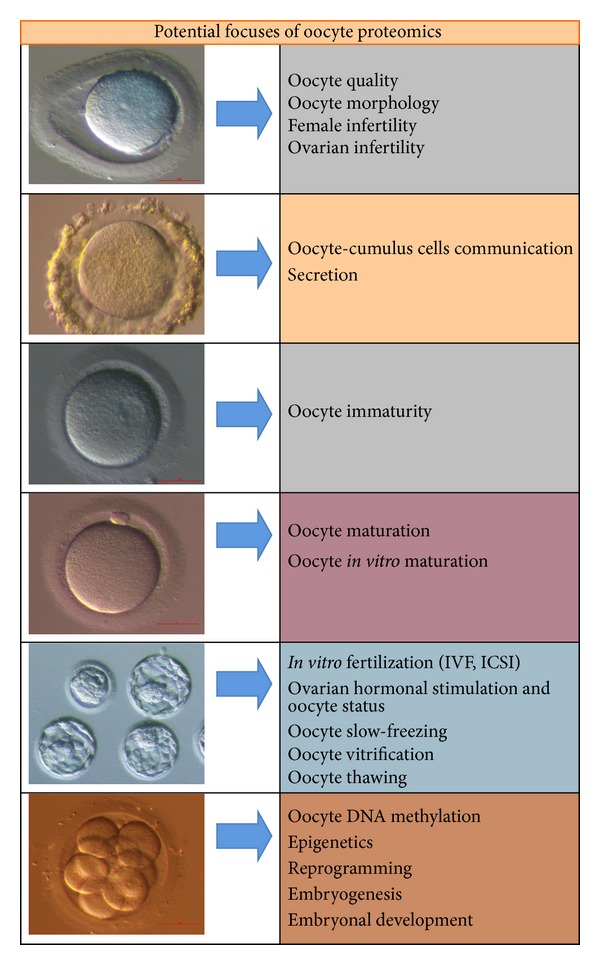
Some potential focuses of proteomics to study human oocytes.

Indeed, proteomic approaches are potentially very powerful to elucidate critical aspects of oocyte development or quality (Figure 1), but this is a largely unexplored territory. Studies over the last ten years have studied oocyte proteomes of various mammalian species, however largely excluding human oocytes due to technical issues (e.g., collection of sufficient number of oocytes), lack of access to cells, or to ethical concerns. The aim of this review is to summarize the literature on animal oocyte proteome and secretome studies to elucidate what can be learned for human oocytes in the* in vitro* fertilization programme.

## 2. Proteomics of Animal Oocytes

Most of the oocyte proteomic studies were performed in the mammalian species, especially in mouse, bovine, and porcine models. In all these studies oocytes were aspirated from ovarian follicles by mostly 18-gauge needle attached to a sterile syringe or vacuum system from the animals with or without pretreatment with gonadotropins. In some studies oocytes were aspirated from isolated ovaries to retrieve sufficient numbers of oocytes to be analyzed. Typically samples consisted of several hundreds to several thousands of oocytes. In some studies the zona pellucida was removed from oocytes by enzyme or acid solution to possibly increase the number of detected proteins in the cytoplasm, nucleus and oolemma but in most studies intact oocytes were analyzed. Despite continued progress in proteomic technologies, in particular mass spectrometry, maximizing the number of available cells is of critical importance to reach sufficient proteome depth. In the study of Wang et al. [13] an impressive number of 7,000 mouse oocytes at different developmental stages were analyzed using semiquantitative mass spectrometry, identifying 2,781 proteins in immature oocytes, 2,973 proteins in mature oocytes, and 2,082 proteins in fertilized oocytes (zygotes). Because of the large number of analyzed oocytes, this study provided a deep insight into the protein expression profile of mouse oocytes. This has demonstrated that oocytes are quite “active” cells expressing proteins related to a range of biological functions such as protein metabolism, transport, cell cycle and proliferation, stress response, developmental processes, RNA and DNA metabolism, cell organization and biogenesis, cell-cell signaling, signal transduction, and cell adhesion [13]. Yet, the number of detected proteins was significantly lower than the number of transcripts (approximately 16,457 genes) in mature mouse oocytes analyzed by SOLiD whole transcriptome analysis [14]. Comparison of protein expression profiles of mouse oocytes to mouse embryonic stem cells (mESCs) showed high similarity between immature and mature oocytes on one hand, and between fertilized oocytes (zygotes) and mESCs on the other hand [13] which may be explained by the activation of the molecular programme in oocytes transiting into the embryo.

In other proteomic studies fewer oocytes were analyzed resulting in lower numbers of detected proteins. Yet, as further discussed below, they have elucidated important relations between oocyte proteome composition and oocyte quality, maturity,* in vitro* maturation, and other* in vitro* fertilization procedures.

## 3. Proteomics and Oocyte Quality

In the* in vitro* fertilization programme, ovaries of women are hormonally stimulated to retrieve more oocytes for fertilization and for treatment of severe infertility. Little is known about oocyte quality admitted in the* in vitro* fertilization programme, where oocyte morphology is almost the only criterion used in daily medical practice. Therefore, in the absence of objective (molecular) criteria for oocyte quality, it is almost impossible to predict the outcome of* in vitro* fertilization treatment. Indeed, much more has been learnt from protein expression profiling either directly from human oocytes or by inferring such insight from animal oocytes.

Among very few studies on this topic, Powell et al. [16] aimed to discover putative quality markers of pig oocytes by comparative proteomics using differential labeling of proteins by ExacTags, followed by quantitative tandem mass spectrometry. Pig oocytes can be obtained in much larger quantities than rodent or nonhuman primate oocytes. In addition, they provide an excellent biomedical model as they mimic some of the events of oocyte maturation and early development in humans. Pig oocyte extracts (oocyte proteome) from groups of 100 oocytes and pig oocyte-conditioned* in vitro* maturation media (oocyte secretome) were obtained from high- and low-quality pig oocytes in terms of their developmental potential in TCM-199 medium with or without added gonadotropins FSH and LH. Sixteen abundant proteins were identified in the oocyte proteome that were differentially expressed in high- and low-quality oocytes (see Table 1). Proteins that were more abundant in the proteome of high-quality oocytes included kelch-like ECH-associated protein 1 (an adaptor for ubiquitin-ligase CUL3), nuclear export factor CRM1 (controlling the movement of DNA methyltransferase 1 (DNMT1) and maintenance of DNA methylation patterns in the early embryo), and ataxia-telangiectasia mutated protein kinase (may be involved in DNA repair). Interestingly, low-quality oocytes secreted monoubiquitin as well as several proteins implicated in human disease, such as dystrophin (DMD) and cystic fibrosis transmembrane conductance regulator (CFTR), two proteins implicated in muscular dystrophy, and cystic fibrosis, respectively. It was concluded that quantitative proteomic analysis of limited samples sizes can suffice to identify potential markers reflecting oocyte quality, which might also be applied for the detection of biomarkers of human oocytes noninvasively.

## 4. Proteomics and Oocyte Maturity 

Oocyte maturation is a complex process consisting of a cascade of molecular events leading to the marking of the following generation, and proper progression through these stages is critical to the success of assisted reproduction techniques (ART) in humans and other mammals [17–21]. In the* in vitro* fertilization programme the majority of oocytes retrieved from ovaries by ultrasound-guided aspiration of follicles is mature, that is, at the metaphase II (MII) stage, and thus can be fertilized. But there is also a proportion of immature oocytes at the prophase I/germinal vesicle (GV) or metaphase I (MI) stage that is refractory to fertilization. Since immature oocytes decrease the female fertility and impair the* in vitro* fertilization outcome, it is important to have insight into oocyte maturity. Proteomic approaches may help to identify proteins that reflect oocyte maturation state with important implications for female meiotic maturation and further embryonic development.

### 4.1. Proteomes of Immature and Mature Oocytes

Proteomic studies of oocytes at different stages of maturation using tandem mass spectrometry revealed the expression of transforming acidic coiled coil containing protein (TACC3) in mouse immature GV-stage oocytes [22]. This protein plays a role in the microtubule-dependent coupling of the nucleus and the centrosome and is a motor spindle protein that may play a role in stabilization of the mitotic/meiotic spindle. It may be involved in the control of cell growth and differentiation and may contribute to cancer. In situ hybridization of mouse ovarian tissue sections displayed abundant expression of TACC3 specifically in the cytoplasm of growing oocytes but not in the primordial or atretic follicles. This pattern of expression indicated that in the mouse TACC3 is expressed in ovarian cells undergoing active growth and development.

On the other hand, proteomics revealed a group of highly abundant heat shock proteins and molecular chaperones in the mature MII-stage mouse oocytes and their localization on the plasma membrane, that is, the oolemma [23]. This suggests that heat shock proteins and molecular chaperones are not only involved in their “usual actions” such as oocyte response to environment by regulation of apoptosis (defense mechanisms) and protein folding but may also play a role in the process of oocyte maturation.

In a comparison of mouse GV and MII-stage oocytes, Vitale et al. [24] used two-dimensional (2D) electrophoresis and mass spectrometry identifying 500 proteins, 12 of which were differentially expressed between these stages (Table 2). Similar to the studies mentioned above these proteins also include TACC3 protein and heat shock proteins (HSP105, stress-inducible phosphoprotein STIP1 that acts as an adaptor protein coordinating the functions of HSP70 and HSP90). Among these proteins were also two epigenetics-related proteins: nucleoplasmin 2 (NPM2; essential for nuclear and nucleolar organization and early embryonic development) and spindlin (SPIN1; a major maternal protein expressed in the mouse during the transition from oocyte to embryo). In addition, it was found that NPM2, SPIN1, programmed cell death six-interacting protein (PDCD6IP), and importin alpha2 may be posttranslationally modified by phosphorylation during oocyte maturation [24]. Prompted by the fact that NPM2 is an oocyte-restricted protein related to epigenetic regulation, further investigations into its properties during oocyte maturation and preimplantation development revealed that NPM2 mRNA levels rapidly decline at fertilization. Indirect immunofluorescence analysis showed that, with the exception of cortical localization in mature MII-stage oocytes, NPM2 is localized in the nucleus of GV-stage oocytes and at all stages of preimplantation embryos.

Cao et al. [25] used a similar approach using 2-DE methodology identifying 63 proteins differentially expressed between mouse GV and MII-stage oocytes after removal of the zona pellucida (see Table 2). Six of these proteins overlapped with those in above mentioned study of Vitale et al. [24] (e.g., NPM2, SPIN1, adenylosuccinate synthetase related to purine biosynthesis, and Nudix). Among differently expressed proteins were 21 proteins which were decreased or even absent in MII-stage oocytes, while 33 proteins were more abundant in mature MII-stage oocytes. Interestingly, three of these proteins were maternal effect proteins related to epigenetics: NPM2, SPIN1, and protein-arginine deiminase type-6 (PADI6). The differently expressed proteins showed significant enrichment for four pathways: purine metabolism, proteasome, glycolysis and gluconeogenesis, and pyruvate metabolism and were related to meiosis, fertilization, and regulation of early embryo development.

Some other studies showed that also protein phosphorylation changed during oocyte maturation. In the study of Ma et al. [26], two-dimensional electrophoresis (2-DE) of mouse MII-stage oocyte proteins and staining with silver staining or Pro-Q Diamond dye was performed to describe the proteome and phosphoproteome of mouse oocytes. A total of 380 unique proteins were identified, in which 90 protein spots representing 53 (14%) unique proteins were stained with Pro-Q Diamond thus suggesting that they were phosphorylated. Moreover, Pelech et al. [27] investigated the regulation of protein kinases, phosphatases, and other regulatory proteins during meiotic maturation of pig immature GV-stage oocytes to maturing MI-stage oocytes and fully mature MII-stage oocytes. Most of the detected changes during the GV to MI transition were related to increased levels of protein kinases, while reduced protein kinase levels and increased protein phosphorylation characterized the MI to MII transition of pig oocytes. Many of the increased protein levels associated with MI were completely or partially reversed during the MI to MII transition. The regulation of these proteins was also confirmed in maturing bovine oocytes [27].

In spite of these and other studies there is still no generally accepted list of proteins related to immature and mature oocytes in mammals, and therefore further relevant studies need to be done. It is encouraging that the reported studies identified an overlapping set of proteins that are differently expressed in immature and mature oocytes (e.g., adenylosuccinate synthetase, STIP1, Nudix, NPM2, and SPIN1). These proteins may represent the crucial proteins related to the oocyte immaturity/maturity and may be interesting for the human reproductive biomedicine in the future. Yet, discordant results between these studies might reflect the fact that oocytes were retrieved from different animal strains and that different proteomic methods were used to elucidate the proteomes. This warrants a more systematic analysis, especially targeted at human oocytes.

### 4.2. Proteomes of Oocytes and Surrounding Cumulus Cells

Oocyte maturation is a highly complex process directed in part by communication between the oocyte and the surrounding follicular cells (cumulus cells). Although the nature of these interactions is largely unknown, cumulus cells are essential for proper maturation of oocytes and further embryonic development upon fertilization. Breakdown of the germinal vesicle (GV) is one of the fundamentals for development and maturation of fully grown, developmentally competent oocytes in mammals. Memili et al. [28] have analyzed the proteomes of five hundred bovine GV-stage oocytes and their surrounding cumulus cells identifying 1,092 proteins in oocytes and 4,395 proteins in the corresponding cumulus cells, 858 of which were in common. This indicates that the oocytes and surrounding cumulus cells are two distinct types of cells sharing a subset of proteins. In further steps Peddinti et al. [29] identified 811 and 1,247 proteins in bovine GV-stage oocytes and corresponding cumulus cells using differential detergent fractionation and multidimensional protein identification technology; 371 proteins were differentially expressed between the two cell types with statistical significance. Further modeling showed that cumulus cells, when compared to GV-stage oocytes, have higher expression levels of proteins involved in cell communication, generation of precursor metabolites and energy, and transport. The authors suggested that oocytes may depend on the presence of cumulus cells to generate specific cellular signals to coordinate their growth and maturation [29].

### 4.3. Oocyte* In Vitro* Maturation at the Proteome Level

*In vitro* maturation (IVM) of oocytes is of big interest in the field of assisted reproduction and infertility treatment. Among oocytes retrieved in the* in vitro* fertilization programme there is a proportion of immature GV and MI-stage oocytes which cannot be fertilized. The possibility has been explored to aspirate these oocytes from the ovaries of women with polycystic ovary syndrome (PCOS) and mature them* in vitro* to prevent ovarian hyperstimulation in these women [30–32]. Alternatively, immature oocytes from fresh ovarian cortex tissue can be stored before chemotherapy and radiotherapy, to preserve fertility in young women with cancer and to mature and fertilize them* in vitro* [33]. In this way autotransplantation of thawed ovarian tissue for fertility restoration would be replaced by* in vitro* fertilization of* in vitro* matured oocytes from the tissue, thus avoiding the risk of retransplantation of malignant cells. In spite of this interest, oocyte* in vitro* maturation is a low-success procedure at present producing oocytes with a poor clinical outcome shown by poor embryo development and early pregnancy loss [34].

A number of proteomic studies have been performed to start understanding oocyte maturation at the molecular level. Berendt et al. [35] used 2-D DIGE saturation labeling approach for quantitative proteome profiling of bovine immature GV-stage oocytes versus* in vitro* matured MII-stage oocytes. Spots of a preparative gel from 2,200 oocytes were identified by nano-LC-MS/MS analysis, 10 of which were differentially expressed between immature and* in vitro* matured bovine oocytes including cell cycle-associated proteins and redox enzyme variants (see Table 3). Similarly, Kim et al. [36] identified proteins that were differently expressed during* in vitro* maturation of porcine oocytes using 2-DE analysis followed by mass spectrometry. They specifically focused on the proteins that were upregulated during the oocyte MII-stage when compared with the GV-stage. Similar to Berendt et al. [35] they found proteins related to the cell cycle, redox regulation (e.g., peroxiredoxin 3 (PRDX3)), and also to the cAMP-dependent pathway, which is essential for the intracellular signaling involved in oocyte maturation (see Table 3). These proteins may be involved in oocyte meiotic resumption, MII arrest, and oocyte activation. Interestingly, the results also indicated that heat shock proteins and zona pellucida glycoproteins may be involved in the oocyte* in vitro* maturation process. The protein candidates identified by these studies may help to improve the oocyte* in vitro* maturation procedure and increase the rate of* in vitro* fertilization and other ART procedures in mammals.

Interestingly, in the discussed studies there was no overlap between differentially expressed proteins in the MII-stage oocytes which matured* in vivo* or* in vitro* in comparison with immature GV-stage oocytes. This raises an important question about the potential molecular differences between* in vitro* and* in vivo* matured MII-stage oocytes which is a crucial issue that needs to be answered in the future. Of note, the expression of several epigenetics-related proteins (e.g., NPM2, SPIN1) were differently expressed between* in vivo* matured oocytes and immature oocytes but were equally expressed in* in vitro* matured oocytes and immature oocytes. This suggests that these proteins are differentially expressed in oocytes matured* in vitro* and* in vivo*.

## 5. Other Procedures of* In Vitro* Fertilization and Proteome

An important part of the* in vitro* fertilization programme is the cryopreservation of oocytes, for example, when an insufficient number of sperm is available on the day of* in vitro* fertilization [37] or for preservation of fertility before chemotherapy and radiotherapy in young women with cancer [38]. Oocytes can be preserved by two different approaches: slow freezing [39] or vitrification [40]. Proteomic studies showed that slow-freezing may affect the proteome of mouse oocytes when compared with control and vitrified mouse oocytes [41]. Similarly, Katz-Jaffe et al. [42] observed that mouse MII-stage oocytes exposed to 1.5 M propandiol (PrOH), a cryoprotectant for slow oocyte freezing, for 20 minutes exhibited significantly altered protein profiles in comparison to controls (11 proteins were downregulated and 8 were upregulated). On the other hand, they found that the temperature changes per se (during cooling to room temperature) have limited effect on the oocyte proteome. From all these data we may conclude that oocyte preservation by vitrification is less deleterious at the proteome level than standard slow-freezing procedure and may therefore be the preferred procedure. These data also indicate that proteome analysis is a powerful approach to evaluate the effect of* in vitro* fertilization procedures on the oocyte molecular status.

Moreover, a study [13] comparing mouse immature, mature, and fertilized oocytes has identified proteins that may be involved in the unique oocyte ability to reprogramme other (sperm or somatic) cell nuclei, and that thus might be very helpful in more efficient creation of induced pluripotent stem cell (iPSCs) lines in the future. The results showed that specific transcription factors and chromatin remodeling factors were more abundant in mature MII-stage oocytes than in immature oocytes. This may be crucial for the epigenetic reprogramming of sperm or somatic nuclei in oocytes.

## 6. Proteins Related to Epigenetics: Maternal Effect Proteins

Among a range of proteins detected by proteomics in different studies it is difficult to prioritize protein groups for functional analysis with respect to oocyte quality and maturation status, and to efficiency of* in vitro* fertilization procedures. Arguably, proteins related to epigenetic regulation represent an important class of proteins impacting on fundamental processes of early human (mammalian) development, that is, imprinting, DNA methylation, embryogenesis, and embryo development on one side and the manifestation of cancer on the other. Altered expression of these proteins may seriously affect development and embryogenesis, possibly resulting in anomalies in a baby. In Table 4 we can see the knockout mouse phenotypes related to decreased fertility, embryo arrest, and even death [43]. Several studies discussed in this paper showed that some epigenetics-related proteins such as nucleoplasmin 2 (NPM2), spindlin 1 (SPIN1), and protein-arginine deiminase type-6 (PADI6) were differently expressed between mature and immature animal oocytes indicating that these proteins are involved in the oocyte maturation process.

**Figure 2:**
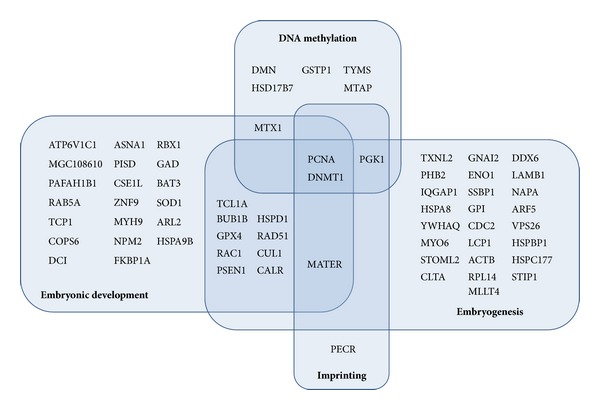
Biological processes (indicated in bold) that are regulated by maternal effect proteins in oocytes, adapted from Zhang et al. [15].

The study of Zhang et al. [15] showed that proteome analysis could be a valuable resource to aid in the characterization of important maternal effect proteins in oocytes, involved in oogenesis, fertilization, early embryonic development, and revealing their molecular mechanisms of action. Using a high-performance proteomic approach they identified 625 different proteins from 2,700 mature mouse oocytes without zona pellucida. Among these proteins they also screened 76 maternal proteins (Figure 2) with high levels of mRNA expression both in oocytes and zygotes, including well-known maternal effect proteins such as MATER and NPM2. Moreover, Yurttas et al. [43] identified in mouse MII-stage oocytes a palette of abundant* bona fide* or potential maternal effect proteins, as can be seen in Table 4. In addition, the authors suggested putative “maternal effect structures” of the mouse MII-stage oocytes that they have predicted to include maternal effect proteins playing a central role in mediating the oocyte-to-embryo transition: cytoplasmic lattices, multivesicular aggregates, spindle apparatus, subcortical maternal complex, endoplasmic reticulum, and microtubule organizing centre.

All these findings are potentially extremely important and would need to be further researched on human oocytes for potential clinical implication. The proteins related to epigenetic regulation are definitely the first choice to evaluate the safety of assisted conception procedures at the oocyte molecular level.

## 7. What about Proteomics of Human Oocytes?

Although we highlighted several proteomic studies focusing on animal oocytes, to our knowledge there is no proteomic data of human oocytes in the literature at present. There are several reasons for this. Human oocytes are sensitive and scarce biological material to be researched; access to them is restricted by several ethical issues. The only potential source of oocytes is those that are discarded in daily medical practice in an* in vitro* fertilization programme, that is, immature oocytes and mature oocytes which do not fertilize after the* in vitro* fertilization procedure. In this way, oocytes can be researched after approval of the medical ethical committee and donated for a research after the donor's written informed consent. An additional issue is the relatively large number of oocytes required for proteomic studies. One possibility to get around this is by stepwise collection of oocytes, their storage in a deep-freezer or liquid nitrogen, and pooling into bigger samples. Also some issues related to cell culture would need to be avoided such as culture of oocytes in usual* in vitro* fertilization media containing high amounts of serum albumin to be able to detect a higher number of proteins by proteomics. Even under optimal conditions oocytes from the* in vitro* fertilization programme may represent only a model for natural human oocytes because they are removed from the ovarian niche, and also the surrounding cumulus cells are denuded during the procedure. In addition, nonfertilized oocytes from the programme might possess increased pathologies at the molecular level. Notwithstanding these obstacles, direct access to human oocytes is of great importance to possibly improve the outcome of* in vitro* fertilization, and therefore the elucidation of the human oocyte proteome remains an important task for the future. Ideally this includes the human oocyte secretome to gain insight in oocyte signalling and functionality.

To avoid the problems related to restricted access to oocytes, human follicular fluid has been proposed as an alternative for indirect evaluation of oocyte quality. Human follicular fluid is a complex fluid that represents the microenvironment of developing oocyte/follicle in the ovary. Follicular fluid supports oocyte maturation and ovulation; however its proteome composition is largely unknown. Potentially, information about its protein constituents may provide a better understanding of ovarian physiology in addition to opening new avenues for investigating ovarian disorders. Follicular fluid is aspirated from the follicles during oocyte retrieval from women undergoing* in vitro* fertilization treatment and is discarded in daily medical practice, and may thus be available for proteome analysis. Indeed, following initial proteomic analyses of porcine [44] and canine [45] follicular fluid, also the proteome of human follicular fluid was reported recently [46–49], detecting up to 480 proteins [49]. The identified proteins belong to different functional categories including growth factors and hormones, receptors, enzymes, and proteins related to defense/immunity and complement activity thus reflecting a very intense cellular activity in the follicle. Interestingly, one study [46] identified potential biomarkers of good versus poor responders to ovarian stimulation in patients included in the* in vitro* fertilization programme. Moreover, they found a group of proteins (e.g., haptoglobin alpha, predominantly fetal expressed T1 domain, and apolipoprotein H) which showed an increased expression in the follicular fluid of “live birth” group of patients. Another study [48] revealed small groups of proteins related to “live birth,” “miscarriage,” and “no pregnancy.” In addition, more proteins related to biosynthesis were found in follicular fluid samples corresponding to the oocytes resulting in pregnancy after* in vitro* fertilization. Notably, there was little overlap among the proteins provided by these studies.

Although the results of these studies seem promising, further research is needed in patient groups, and proteomes need to be sampled at greater depth. Until then, the reported results need to be interpreted with caution. Clearly, and as with any clinical study, proteome analysis of follicular fluid remains a difficult task due to potential variation introduced by several factors, for example, age, ovarian pathologies and general health status, and the type of ovarian hormonal stimulation.

## 8. Future Perspectives of Human Oocyte Proteomics

As evidenced by the body of literature discussed above, application of proteomic technologies has made important contributions to gaining insight in proteome composition of oocytes and changes therein during development or in procedures related to assisted reproduction. This is particularly true for animal cells, mainly for reasons related to availability of cells. Although animal studies can be very insightful where many questions can be answered to uncover fundamental aspects of oocyte biology, it is clear that attention needs to be turned to human oocytes to make an impact on natural and assisted human reproduction. One of the major bottlenecks is the scarce amount of sample that can typically be obtained, and therefore it is of crucial importance to deploy the most sensitive proteomics technologies and to keep investing in the development of such methodologies. As is apparent from the studies discussed here, 2D-gels have been the prevalent technique for proteome profiling. Because of the limitations of 2D-gels in overall sensitivity, in displaying very large or very small proteins, as well as hydrophobic proteins (including membrane proteins), they have been largely replaced by LC-based (multidimensional) peptide separation. Adoption of proteome analysis by LC-MSMS leads to higher numbers of identified proteins and requires less sample input. Importantly, implementation of miniaturized peptide separation platforms coupled to sensitive and high-resolution mass spectrometry allows for in-depth proteome profiling even for rare cell types [50]. Furthermore, accurate protein quantification is essential when aiming to identify proteins that differ in expression as a result of developmental progression, cell preservation techniques, or patient treatment. To this end, approaches using stable isotope labeling are abundant in the proteomics field, including developmental biology [51], but, with very exceptions, have not permeated in the area of oocyte biology yet. Implementation of these approaches, along with continued developments in sample preparation and mass spectrometric technologies, should enhance progress in this field. Envisioning that sample amounts in the low-*μ*g range may suffice in the foreseeable future; a few dozen (or fewer) oocytes should be sufficient for in-depth and quantitative proteome analysis, thus making this an accessible route for scarce human oocytes. This view may be inspired by developments in next-generation sequencing technologies now capable of sequencing genomes of single human oocytes [52], which was unimaginable just a few years ago.

## 9. Conclusion

With advanced proteomic technologies in place, many questions remain to be answered related to oocyte quality, maturation, and sensitivity to procedures of assisted conception. The picture that is emerging from the animal studies discussed above is that a relatively small group of proteins differ in expression between oocytes in different quality or maturation groups. Furthermore, it is encouraging that there is considerable overlap between these proteins from different studies in spite of the heterogeneity of animal species and strains, and in the methodology used. This suggests that these differences are robust, and that they may also apply to human oocytes. Maybe most interestingly, some epigenetics-related proteins were found to be differently expressed during oocyte maturation, suggesting that they deserve closer attention in the context of human reproductive medicine. We have provided some lists of additional proteins from the literature that might be of further interest to the study of human oocytes. Despite their important function in oocyte biology, secreted proteins are poorly covered in these studies, although secretome strategies are beginning to emerge [16, 53] for animal models. Extension of such studies to human oocytes could provide important cues reflecting oocyte functionality, particularly in response to different conditions. Alternatively, follicular fluid from patients who undergo* in vitro* fertilization seems to be an interesting source of informative biomolecules. We strongly believe that combined efforts between* in vitro* fertilization programmes and labs specializing in proteomics will create the conditions to have access to valuable patient material for meaningful analysis by advanced and sensitive proteomic technologies. This should provide fundamental insight into the earliest stages of human life and may come to the benefit for those who need to rely on assisted reproduction to get a baby.

## Figures and Tables

**Table 1:** Proteins that are differently expressed in the proteomes of high- and low-quality pig oocytes [16].

Differently expressed proteins in low- and high-quality pig oocytes	
Upregulated in high-quality oocytes	
Glutaminase (GLS)	
Hypothetical protein Gi/24528344	
Nuclear export factor CRM1 (CRM1)	
Kelch-like ECH-associated protein 1 (KEAP1)	
IS10-right transposase	
Albumin	
Ryanodine receptor (RyR)	
Cortactin-binding protein 2 (CTTNBP2)	
Fatty acid synthase (FASN)	
Epidermal growth factor (EGF)-receptor (EGFR)	
Ataxia-telangiectasia mutated protein (ATM)	
Upregulated in low-quality oocytes	
*β*-1 adrenergic receptor (ADRB1)	
25-Hydroxyivitamin D3 1*α*-hydroxylase	
Connective tissue growth factor (CTGF)	
Dystrophin (DMD)	
Mannose-6-phosphatase/IGF II receptor	

**Table 2:** Selected proteins that are differently expressed in immature and mature mouse oocytes according to [24, 25].

Some of proteins differently expressed in immature and mature mouse oocytes	
Vitale et al. [24]:	
Transforming acidic coiled coil containing protein (TACC3)	
Heat shock protein 105 (HSP105)	
Programmed cell death six-interacting protein (PDCD6IP)	
Stress-inducible phosphoprotein (STIP1)	
Importin alpha-2 (KPNA2)	
Adenylosuccinate synthase (ADDS)	
Nudix	
Spindlin (SPIN)*	
Lipocalin (LCN)	
Lysozyme	
Translationally controlled tumor protein (TCTP)	
Nucleoplasmin 2 (NPM2)*	
Cao et al. [25]:	
Methionine adenosyltransferase II beta (MAT2B)	
Proteasome (prosome, macropain) 26S subunit ATPase 6 (PSMC6)	
Inositol-3-phosphate synthase 1 (ISYNA1)	
Eukaryotic translation initiation factor 5A (EIF5A)	
Protein-arginine deiminase type-6 (PADI6)*	
CTP synthase (CTPS)	
Adenylosuccinate synthase (ADDS)	
Nucleoplasmin 2 (NPM2)*	
Zinc finger, BED-type containing 3 (ZBED3)	
Lambda-crystallin homolog (CRYL1)	
Guanine deaminase (GDA)	
Insulin-like growth factor 2 mRNA binding protein 2 (IGF2BP2)	
Nudix (nucleoside diphosphate linked moiety X)-type motif 5 (NUDT5)	
Spindlin 1 (SPIN1)*	

Legend: *highly important (maternal effect proteins related to epigenetics).

**Table 3:** Proteins that are differently expressed in immature and *in vitro* matured mammalian oocytes according to [35, 36].

Proteins differently expressed in immature and *in vitro* matured oocytes	
Berendt et al. [35]	
Ca2+-binding protein translationally controlled tumor protein	
(TCTP)	
Enzymes of the Krebs and pentose phosphate cycles	
Clusterin (CLU)	
14-3-3 epsilon (YWHAE)	
Elongation factor-1 gamma (EEF1G)	
Polymorphic forms of GST Mu 5	
Peroxiredoxin-3 (PRDX3)	
Kim et al. [36]	
Downregulated proteins in IVM oocytes	
Similar to G patch domain and KOW motifs, partial	
Heat shock 70 kDa protein 5/glucose-regulated protein	
(HSPA5)	
Precursor (GRP78) isoform 1	
TD and POZ domain-containing protein 1 (TDPOZ1)	
Protein disulfide isomerase/protein disulfide-isomerase A3 precursor (ERP57)	
M-phase phosphoprotein 1 (MPP1)	
Chain A, Steric and Conformational Features of the aconitase mechanism	
Zona pellucida sperm-binding protein 3 precursor (ZP3)	
Zona pellucida glycoprotein 4 (ZP4)	
Cerebellar degeneration-related protein 2 (CDR2)	
Peroxiredoxin 3 (PRDX3)	
Heat shock protein 27 kDa (HSP27)	
Upregulated proteins	
Protein kinase 5′-AMP-activated protein kinase subunit beta-1 (PRKAB1)	
Myomegalin phosphodiesterase 4D interacting protein	
(PDE4DIP)	
Major vault protein similar to lung resistance-related protein homologue (MVP)	
Heat shock protein HSP 90-alpha 2 (HSP90AA2)	
Heat-shock protein hsp86 (HSP86)	
Heat shock protein 70.2 (HSP70.2)	
Phosphoglucomutase 5 (PGM5)	
Dystrobrevin alpha (DTNA)	
Cytoskeletal beta actin (ACTB)	
Spermine synthase (SPSY)	
Galactokinase 1 (GALK1)	
Transferase, HG-phosphoribosyl (HGPRT)	
Glutathione S-transferase, mu 2 (GSTM2)	
Glutathione-S-transferase, mu 5 (GSTM5)	
Peroxiredoxin-2 (thioredoxin peroxidase 1) (PRDX2)	

**Table 4:** *Bona fide* and putative maternal effect proteins (genes) identified in proteomic screens of mouse MII oocytes according to Yurttas et al. [43].

Maternal effect protein (gene)	Knockout mouse phenotype	Localization within oocyte	Association with maternal effect structures (MESs)
*Bona fide* maternal effect proteins			
DNA methyltransferase (cytosine-5) 1 (*Dnmt1*)	Death at E14 to E21	Cytoplasmic	Maybe CPL, MVA or SCMC
FILIA (*2410004A20Rik*)	50% decrease in female fertility	Cortex	SCMC
Factor located in oocytes permitting embryonic development—FLOPED (*Ooep*)	Two- to four-cell embryo arrest	Cortex	SCMC
Maternal antigen the embryos require—MATER (*Nlrp5*)	Two-cell embryo arrest	Cortex	SCMC
Nucleoplasmin 2 (*Npm2*)	70% decrease in female fertility, 95% of embryos arrest before blastocyst stage	Nuclear, transient cortical staining during the MII oocyte stage	Maybe SCMC
2′–5′ oligoadenylate synthetase 1d—OAS1D (*Oas1d*)	30% decrease in female fertility, 40% of embryos arrest by eight-cell stage	Cytoplasm and cortex	Maybe CPL, MVA, or SCMC
Peptidylarginine deiminase 6 (*Padi6*)	Two-cell embryo arrest	Cytoplasm and cortex	CPL, maybe SCMC
Putative maternal effect proteins			
Cytosolic phospholipase A2*γ* (*Pla2g4c*)	NA	Cytoplasm and cortex	Spindle, maybe MVA, or SCMC
Developmental pluripotency-associated 5A—DPPA5A (*Dppa5a*)	Normal fertility due to possible redundancy with KH family members	Cytoplasm	Maybe CPL or MVA
NLR family, pyrin domain-containing 14—NLRP14 (*Nlrp14*)	NA	Cytoplasm	Maybe CPL or MVA
Spindlin (*Spin1*)	NA	Cytoplasm	Spindle, maybe CPL or MVA
Transducin-like enhancer of split 6 (*Tle6*)	NA	Cortex	SCMC

Legend: CPL: cytoplasmic lattice; SCMC: subcortical maternal complex; MVA: multivesicular aggregate; NA: not available (unknown).
